# SwedeAmp—the Swedish Amputation and Prosthetics Registry: 8-year data on 5762 patients with lower limb amputation show sex differences in amputation level and in patient-reported outcome

**DOI:** 10.1080/17453674.2020.1756101

**Published:** 2020-04-22

**Authors:** Ilka Kamrad, Bengt Söderberg, Hedvig Örneholm, Kerstin Hagberg

**Affiliations:** a Departments of Orthopedics and Clinical Sciences, Lund University and Skåne University Hospital Malmö;; b Department of Orthopedics, Skåne University Hospital Lund;; c Centre for Advanced Reconstruction of Extremities and Department for Prosthetics and Orthotics, Sahlgrenska University Hospital, Gothenburg, and Institute of Clinical Sciences, Department of Orthopedics, Sahlgrenska Academy, Gothenburg, Sweden

## Abstract

Background and purpose — For want of national guidelines for lower limb amputation (LLA) the quality registry SwedeAmp was started in 2011 to increase knowledge around LLA and prosthetic rehabilitation. We now present data from the first 8 years of registration.

Patients and methods — We present descriptive data from the first 8 years (2011–2018) of registration. Patient-reported outcome was collected at baseline and at follow-up 12 and 24 months after surgery for patients with prosthetic supply and included generic (EQ-5D-5L) and amputee-specific (e.g., LCI-5L and Prosthetic Use Score) measures. Sex differences were investigated.

Results — As at December 31, 2018, 5,762 patients, 7,776 amputations, 2,658 prosthetic supplies, 1,848 baselines, and 2,006 follow-ups were registered. 61% of the patients were male, and mean age by the time of the first registered amputation was 74 years (SD 14). Women were older, more frequently had vascular disease without diabetes and more often underwent amputation at a higher level compared with men (p < 0.001). Time from amputation to fitting of first individual prosthesis was median 69 days (6–500) after transtibial amputation (TTA) and 97 days (19–484) after transfemoral amputation (TFA). The outcomes were lower after TFA than after TTA.

Interpretation — SwedeAmp shows sex differences concerning amputation level, diagnosis, and age, leading to the conclusion that women have worse preconditions for successful prosthetic mobility after LLA. With increasing coverage, SwedeAmp can provide deeper knowledge with regard to patients undergoing LLA in Sweden.

Lower limb amputation (LLA) is often discussed from specific points of view such as amputation incidence (Johannesson et al. 2008, Buckley et al. 2012, Fortington et al. 2013b, Jones et al. 2013), mortality (Fortington et al. 2013a, Jones et al. 2013), prosthetic prescription, mobility, and patient-reported outcome (Raichle et al. 2008, Norvell et al. 2011, Davie-Smith et al. 2017a). Moreover, most of those studies include only patients from one hospital or region. The diversity of data and the difficulty of comparing results have been discussed repeatedly (Ephraim et al. 2003, Sinha and Van Den Heuvel 2011, Fortington et al. 2012, Samuelsson et al. 2012, van Netten et al. 2016, Davie-Smith et al. 2017a, b).

According to the Swedish National Board of Health and Welfare, the incidence of major (transtibial level or more proximal) LLA in Sweden, including revisions and re-amputations, has for many decades been between 33 and 39/100,000 inhabitants, with yearly and regional variations from 9–107/100,000. Amputation level, pre- and postoperative care, rehabilitation, and prosthetic supply differ between regions and hospitals. With the intention to provide equal and best possible care for patients with LLA, the Swedish Amputation and Prosthetics Registry for the lower extremity (SwedeAmp) was founded in 2011.

The aim of SwedeAmp is to evaluate the entire medical process regarding LLA in Sweden (Figure 1). We present descriptive data on the first 8 years of registration, evaluate the outcome at 12- and 24-months’ follow-up, and investigate possible sex differences with a focus on major amputations. 

## Patients and methods

SwedeAmp includes patient-based data regarding amputation surgery, prosthetic supply, the patient’s situation, and mobility before amputation and outcome 6, 12, and 24 months after the amputation. Data are registered in 6 different forms (Table 1, see Supplementary data). Forms 1 and 2 include all levels of LLA from partial toe amputation to hemipelvectomy, Forms 3–6 solely major amputations at or proximal to transtibial amputation (TTA) level. The registry uses 3 definitions for surgical procedures: primary amputation, re-amputation, and revision. A revision is defined as a soft tissue revision and/or bone-shortening procedure performed within the same amputation level. Re-amputation is defined as a second procedure on an unhealed residual limb leading to a higher classified amputation level, e.g., from TTA to knee disarticulation (KD) or transfemoral amputation (TFA). Primary amputation is defined as amputation not matching the criteria for revision or re-amputation. Amputation on a higher classified level after previous healed amputation is considered to be a new primary amputation. Furthermore, bilateral amputation is defined in the registry as amputation at/or proximal to the tarsometatarsal level on both sides, performed simultaneously or at different times.

**Table 2.** Underlying diagnosis leading to lower limb amputation and sex differences. Values are number (%)

	Total	Women	Men
Underlying diagnosis	n = 5,544	n = 2,194	n = 3,350
Diabetes with or without			
vascular disease	2,475 (45)	779 (36)	1,696 (51)
Vascular disease without			
diabetes	1,909 (34)	955 (44)	954 (28)
All other diagnoses	1,160 (	460 (20)	700 (21)
Infection not related to			
diabetes or vascular			
disease	207 (4)		
Trauma	215 (4)		
Other (e.g., tumor,			
congenital or acquired			
deformity)	367 (7)		
Diagnosis unknown or			
not registered	371 (7)		

There were statistically significant differences between men and women when underlying diagnosis leading to amputation was grouped as following: diabetes with/without vascular disease, vascular disease without diabetes, and all other diagnoses, p < 0.0001.

To estimate the survival of the patients registered, we used dates of death from the Swedish National Board of Health and Welfare.

For the evaluation of functionality and quality of life, several validated tests and scores are performed at baseline and/or follow-up. The Locomotor Capability Index-5Level (LCI-5L) measures self-reported mobility with a prosthesis (Franchignoni et al. 2004, Larsson et al. 2009). The LCI-5L basic score (values 0–28) and advanced score (0–28) are reported separately and the sum of the 2 scores results in the LCI-5L total score (0–best possible 56). LCI-5L can be used for mobility assessment prior to amputation simply by removing the word prosthesis. The Prosthetic Use Score combines the number of days/week and the number of hours/day the prosthesis is used. A score of 0 indicates that the prosthesis is not used any day/week while a score of 100 indicates wear of prosthesis 7 days/week and more than 15 hours/day (Hagberg et al. 2004). The EQ-5D-5L index estimates the patient’s general health with scores between -0.594 and 1 (full health) (www.euroqol.com). The Timed-Up-and-Go Test (TUG) (Schoppen et al. 2003) assesses functionality and falling risk.

SwedeAmp aims to involve all key professions within the multidisciplinary team. The medical and surgical data are preferably registered by a surgeon, the prosthetic supply by a certified prosthetist and orthotist (CPO), the baseline and follow-up data by a rehabilitation therapist, and the gait data by a CPO or a physiotherapist. SwedeAmp has not yet gained full coverage in Sweden. Figure 2 shows the annual total registrations from 2012 to 2018. In 2018, 11 of the 21 Swedish regions were registered in SwedeAmp. Among these, the coverage for the most common level of amputation, TTA, was 62% compared with data from the National Board of Health and Welfare in 2017 (www.socialstyrelsen.se).

**Figure 1.**
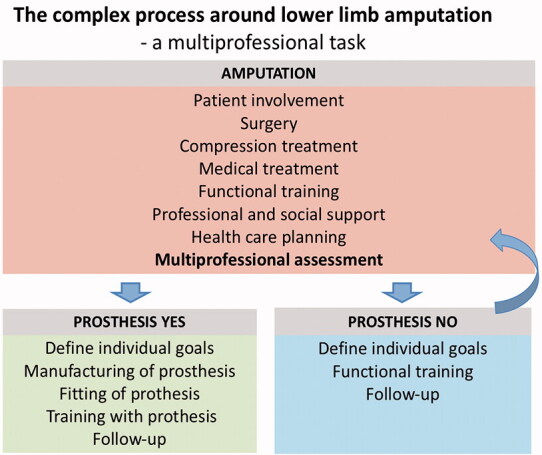
The complex process around lower limb amputation.

**Figure 2.**
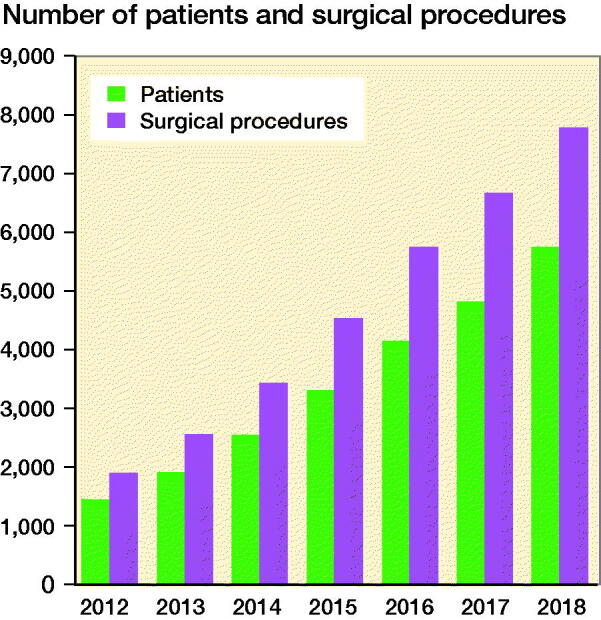
Development of the SwedeAmp registry showing the total registrations in number of patients and surgical procedures from 2012 to 2018.

### Statistics

Data are presented as numbers (n) and %. For continuous data means (SD) are presented and for ordinal data median (md) and min–max values. 95% confidence interval (CI) was reported for mean age at the time of the first registered amputation. To estimate statistically significant differences between groups, chi-square and Mann–Whitney U tests were performed. A p-value of < 0.05 was considered statistically significant.

#### Ethics, funding, and potential conflicts of interest

In accordance with the rules for Swedish national quality registries, patients are informed of registrations in quality registries and have the possibility to decline participation at any time, but no signed consent is needed. This report is based on descriptive data from the open source SwedeAmp report 2018 (www.swedeamp.com) and did not require ethical approval. Funding was received from ALF Skåne and FoU Skåne and the Swedish Government research grant. There are no conflicts of interest.

## Results

### Patients

**Figure 3.**
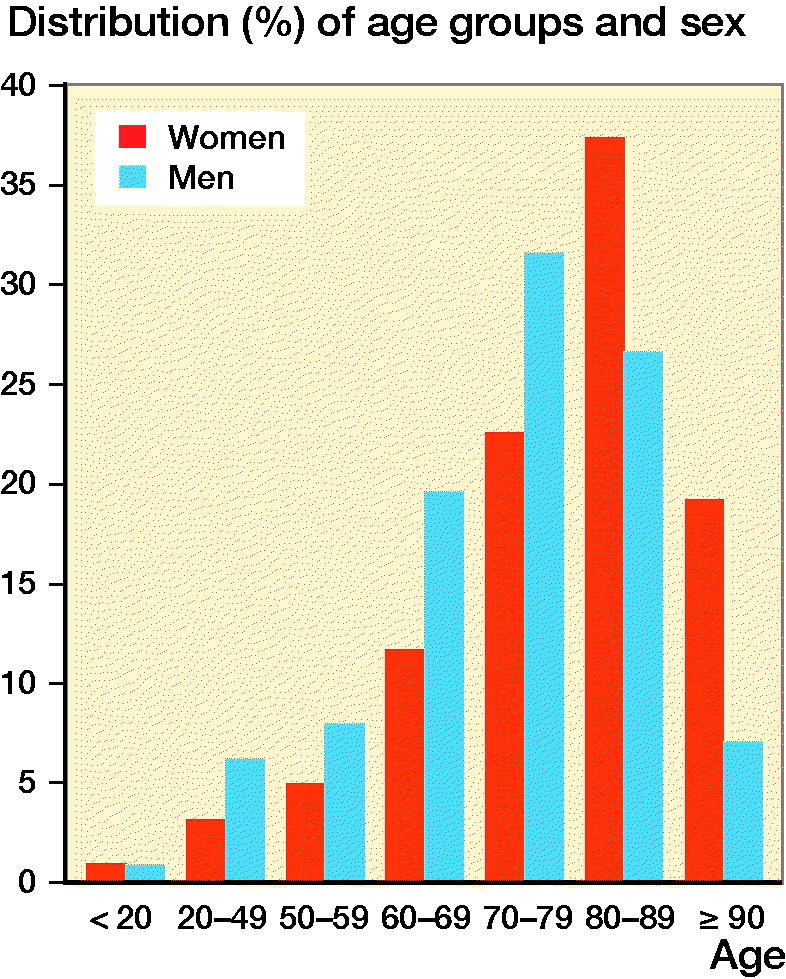
Age and sex at the time of the first registered amputation.

As at December 31, 2018, 5,762 patients, 7,776 amputations, 2,658 prosthetic supplies, 1,848 baseline, and 2,006 follow-up registrations were registered in SwedeAmp. 61% of the patients were male. Mean age at the time of the first registered amputation in our sample was 74 years (SD 14); women were older (78, SD 14, CI 77–79) than men (72, SD 14, CI 72–73). 43% of the patients were 80 years or older by the time of the primary amputation (Figure 3). The mortality rate of the registered patients was 19% within 6 months and 24% within the 1st year after the last registered amputation. The 1-year mortality rate after TFA was 40%, after KD 38%, and after TTA 24%. In 85% of the patients with a registered primary diagnosis, amputation was due to diabetes and/or vascular disease (Table 2). Amputation due to vascular disease without diabetes was reported in 45% of the female patients and in 32% of the male patients (p < 0.0001). 93% of the patients had at least 1 comorbidity, of which the most common were heart disease, lung disease, neurological disease, stroke, or dementia.

Smoking habits were registered for 2,315 patients. At the time of amputation, 39% were non- smokers, 35% previous smokers (not smoked within the last year), 24% current smokers, and 2% were consuming other nicotine products.

#### Amputation data

**Figure 4.**
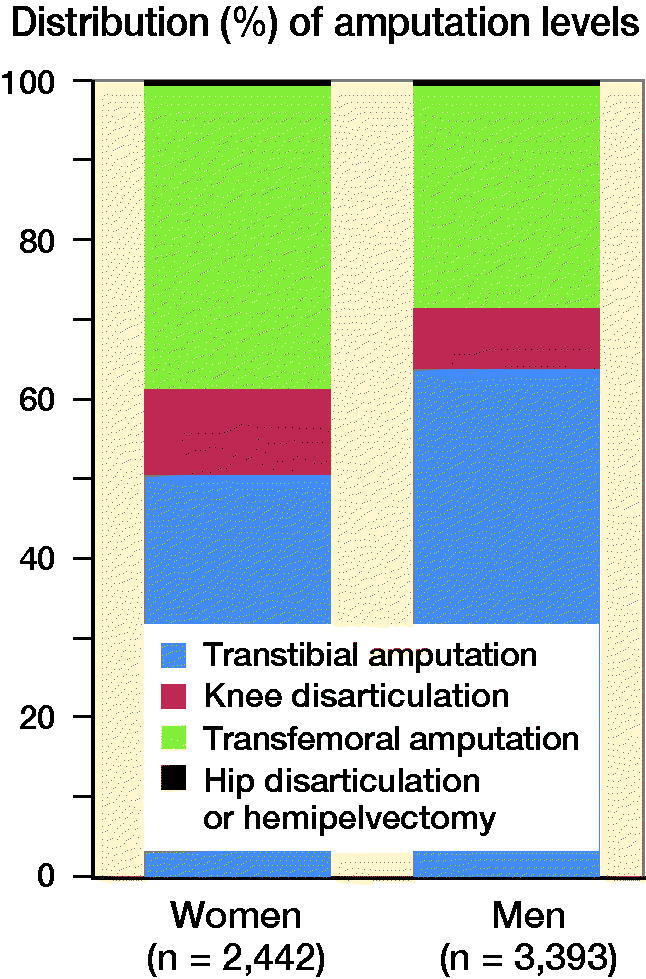
Differences in major amputation levels for men and women (p < 0 .001).

89% of the registrations were unilateral amputations and 80% were primary ones. 14% were re-amputations to a more proximal level and 6% revisions at the same level. TTA was most common (47%) followed by TFA (26%), minor amputation (partial foot amputation distally to ankle level) (20%), KD (7%), hip disarticulation, or hemipelvectomy (< 1%). Figure 4 shows sex differences regarding major amputation levels. 10% of the patients with primary TTA underwent re-amputation to a more proximal level.

The most frequently registered surgical technique for TTA was sagittal flaps (72%) followed by anterior/posterior flaps (14%), long posterior flaps (9%), and skew flaps (4%). Regional differences were seen when considering the use of sagittal flaps, ranging from 33% to 85%. Primary skin closure was performed with sutures in 67% of our cases, with staples in 21%, and open treatment was registered in 2%. In 10% of cases, negative pressure wound therapy was applied additionally. Postoperative residual limb care after TTA included in 95% of the cases a rigid dressing followed by compression treatment with a silicone liner, sometimes combined with an elastic stump shrinker. Liner therapy was in 79% of cases started within 3 weeks postoperatively.

Of the 618 patients registered with bilateral amputations, 68% had TTA at least on 1 side.

Information on antibiotic prophylaxis has been added recently, and of the registered 1,385 cases, no antibiotics were given in 2%, 25% received peroperative prophylaxis, 6% postoperative, and 67% per- and postoperative treatment.

#### Prosthetic supply and self-reported prosthetic use

Postoperatively, 55% of patients with TTA, 25% with KD, and 21% with TFA were assessed as potential users of a functional prosthesis. Of the 2,652 registered prosthetic supplies, 79% were TTA prostheses. The most common type of TTA prostheses included a liner, had vacuum suspension (71%), and an energy-storing foot (79%). TFA prostheses included a large variation of different prosthetic knee components, among which 40% were more advanced knee components such as pneumatic, hydraulic, and/or microprocessor-controlled knee units.

Time from final-level amputation to fitting of the first individual TTA prosthesis was md 69 days (6–500, n = 837) and for TFA 97 days (19–484, n = 158), showing for TTA a decrease over time from 79 days during the first years of registration (2011–2013) to 56 days in 2017–2018. Time from surgery to start of TTA prosthetic rehabilitation was md 82 days (5–484, n = 766) and 112 days (19–490, n = 165) after TFA. Even here a decrease in time to TTA prosthetic rehabilitation could be stated, from md 87 days during 2011–2013 to 64 days in 2017–2018.

Table 3 illustrates the Prosthetic Use Score for patients after unilateral TTA and TFA at 12- and 24-months’ follow-up, respectively. In patients with unilateral TTA due to diabetes and/or vascular disease the mean time to perform the TUG test at 12 months was 26 seconds (SD 19, n = 159) and at 24 months 24 seconds (SD 17, n = 74). In patients with unilateral TTA due to other diagnoses the TUG was 17 seconds (SD 9, n = 54) and 16 seconds (SD 11, n = 23) at 12 and 24 months, respectively.

**Table 3.** Prosthetic Use score and LCI-5L score at 12- and 24-months’ follow-up for patients after unilateral transtibial amputation and transfemoral amputation depending on underlying diagnosis

	Prosthetic Use score **^a^**		LCI-5L Total score **^b^**	
	12 months	24 months	12 months	24 months
DIagnosis	n mean (SD)	n mean (SD)	n median (range)	n median (range)
Transtibial amputation				
Diabetes and/or vascular disease	407 43 (32)	193 55 (33)	458 33 (0–56)	149 37 (0–56)
All other diagnoses **^c^**	83 51 (33)	51 60 (36)	77 43 (3–56)	44 45 (1–56)
Transfemoral amputation				
Diabetes and/or vascular disease	74 16 (21)	35 16 (23)	63 16 (0–56)	25 22 (0–52)
All other diagnoses **^c^**	49 28 (32)	33 34 (35)	46 34 (1–56)	22 43 (8–56)

**^a^**Prosthetic Use score: score 0–100 (= best possible).

**^b^**Locomotor Capability Index–5L: score 0–56 (= best possible).

**^c^**Includes, e.g., trauma, tumor, infection, and not-specified diagnoses.

#### Baseline and follow-up patient-reported outcome

LCI-5L total score prior to amputation was md 43 (0–56, n = 1505), and lower for women (md 36, n = 555) than for men (md 47, n = 950) (p < 0.001). At follow-up LCI-5L scores decreased (Table 3), with lower LCI advanced scores involving more demanding abilities such as walking while carrying an object, walking on stairs without a handrail, or getting up from the floor. After both TTA and TFA, the LCI-5L scores were higher in patients with amputations due to reasons other than diabetes and/or vascular disease.

**Figure 5.**
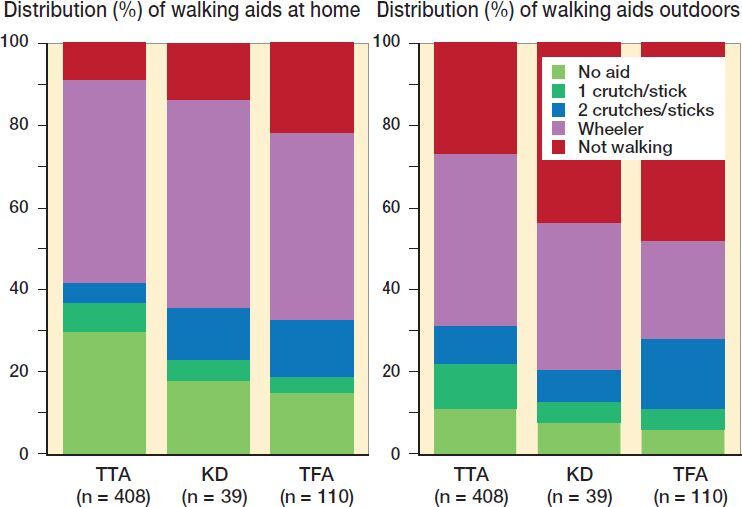
Use of walking aids when walking with the prosthesis at home (left panel) and outdoors (right panel) at 12-months’ follow-up after unilateral amputation.

At follow-up, 81% of the registered patients had returned to the same kind of accommodation as before amputation. About half of our patients used walking aids prior to amputation, and around one-third additionally used a wheelchair. At 12 months’ follow-up most patients used some kind of walking aid together with the prosthesis both at home and outdoors (Figure 5). In addition, > 80% used a wheelchair. Patients with TFA more often than TTA patients reported not walking at all.

At 12- and 24-months’ follow-up, residual limb pain was experienced by 44% and 48% of our patients, respectively, and phantom limb pain by 73% and 69%.

The EQ-5D-5L index in our sample at 12 months was mean 0.57 (SD 0.3, n = 188) and at 24 months 0.56 (SD 0.32, n = 113) in patients with unilateral TTA, and 0.47 (SD 0.36, n = 59) and 0.51 (SD 0.37, n = 43) respectively in patients with unilateral KD or TFA.

## Discussion

To our knowledge, SwedeAmp is the first national quality registry that provides nationwide data on patients undergoing LLA including surgical facts, rehabilitation outcome, and details on prosthetic supply.

Our data confirm LLA patients in the industrialized world to be old and fragile (Dillingham et al. 2002) with high morbidity and mortality rates.

Mobility has previously been reported to be related to satisfaction and quality of life for patients after major LLA (Sinha et al. 2011). Our data showed that the use of walking aids and wheelchair were mandatory for the majority of our patients 1 year after amputation. Moreover, depending on amputation level, not walking outdoors was reported among 27–48% of patients, which increases isolation and dependency. In SwedeAmp most patients underwent unilateral TTA as a result of diabetes and/or vascular disease. This group showed an increased falling risk as the mean TUG time was beyond the 19 seconds Dite et al. (2007) reported to indicate a risk of falling for patients with LLA. Our results emphasize the importance of adequate prosthetic rehabilitation and provision of other assistive devices for patients with major LLA to regain mobility.

SwedeAmp reports females to be older than males by the time of the first registered amputation. Moreover, women were diagnosed more often with vascular disease without diabetes and, probably as a consequence, underwent TFA more often compared with men. This is in line with previous literature (Singh et al. 2008, Davie-Smith et al. 2017b). A possible cause might be the protective effect of estrogen against atherosclerosis before menopause (Vavra and Kibbe 2009, Boese et. al 2017) and thus women may develop vascular disease and related complications later in life. In addition to sex differences, TFA patients scored lower than TTA patients in LCI-5L, Prosthetic Use Score, and EQ-5D-5L. In conclusion, according to our patient sample, female patients in Sweden seem to have worse preconditions for regaining mobility, independence, and general health due to the higher incidence of older age at the time of amputation in combination with the loss of the knee. On the other hand, hypothetically, women might have had a healthier life for longer than men previous to the amputation.

The optimal amputation level is difficult to define. With regard to lower mobility scores and limited use of prostheses after KD/TFA compared with TTA, the surgical aim should be to save the knee (Sansam et al. 2009). On the other hand, vascular impairment is usually worse below the knee level, resulting in an increased risk of revision or re-amputation after TTA compared with TFA. In this sample 10% of the primary TTA patients underwent re-amputation to a more proximal level. Moxey et al. (2010) reported that only 3 of 10 regions in England managed to achieve a TTA/TFA rate greater than 1, a figure which the authors stated to be a quality mark of amputation care.

Residual limb pain and phantom limb pain are common after LLA with incidences up to 70% (Ehde et al. 2000, Morgan et al. 2017). SwedeAmp reports that over 40% of patients experience residual limb pain and about 70% phantom limb pain at least to some degree, without reduction over time. Optimal pain control and surgical technique should be sought to prevent long-lasting pain problems.

In Sweden the use of a postoperative liner for residual limb compression is standard in the postoperative care after TTA (Johannesson et al. 2004). Liner compression was commonly started within 3 weeks after surgery. Regardless, median time from TTA to first fitting of prosthesis was 10 weeks, and a delay of a further 2 weeks was seen until prosthetic rehabilitation started. The cause for this delay cannot be identified by our data. However, the time to the first prosthesis in our material is clearly shorter than the mean of 145 days reported from the United States (Resnik and Borgia 2015). A positive trend could be noted in the registry with decreasing numbers of days to first TTA prosthesis, from md 79 days during the first years of registration (year 2011–2013) to md 56 days (year 2017–2018). With regard to the time from TTA to first fitting of individual prosthesis, the shortest time record (6 days) may be explained by the use of methods involving a laminated socket being produced directly on the residual limb, allowing the start of prosthetic use even before wound healing. However, until now, specific registration of methods involving a socket being directly fitted to the residual limb has not been included in the registry and therefore it cannot be excluded that this short time interval was due to misinput.

EQ-5D-5L is a frequently used index to estimate a patient’s general health; however, it is sparsely used in LLA research. The average EQ-5D-5L index at 12- and 24-months’ follow-up was between 0.47 and 0.56. For comparison, patients undergoing orthopedic surgery in general increase from a preoperative mean of 0.54 to a postoperative mean of 0.72 (Jansson and Granath 2011) and patients with acute coronary syndromes score 0.82 one year after treatment (Gencer et al. 2016). The low scores of amputees show the general morbidity of these patients, and the mortality rate of our patients of 24% within the first year after amputation supports this.

### Limitations

This study is a retrospective registry study and the available data were limited. Interesting facts such as decision-making on the amputation level cannot be detected by the registry. SwedeAmp has not yet gained full coverage and, thus far, our data cannot be taken as representative for Sweden. In some regions, registration of surgical data was done at rehabilitation units with the consequence that only patients who have proceeded to prosthetic rehabilitation are registered from those regions. Thus, surgical data should be interpreted with caution and registration of minor amputations, re-amputations, and revisions is probably underreported. Moreover, mortality and outcome can be considered to represent the best possible indications, as patients not attending prosthetic rehabilitation are so far underrepresented in the registry. Therefore, we cannot make any statement on how patients never reaching prosthetic supply rate their quality of life. Our study only sparsely presents the separate outcome for patients with amputations due to diagnoses other than diabetes and/or vascular disease or for patients with bilateral amputations, due to small numbers in these sub-groups to date. SwedeAmp is continuously being adapted and several variables have been added more recently, e.g., several surgical details such as antibiotic treatment and the prosthetic comfort score (Hanspal et al. 2003). Not all variables are mandatory, leading to different numbers depending on the variable.

## Conclusion

We found worse functional outcome after TFA compared with TTA. Female patients were older by the time of amputation and amputation was performed at a higher level. Time from amputation to prosthetic supply and training has decreased during the most recent years. The results give a general insight into the patient group (dominated by the frail elderly) and the outcomes after major amputation.

With increasing coverage, SwedeAmp may provide deeper knowledge with regard to patients undergoing LLA in Sweden and help to identify associations between the patient’s preoperative preconditions, surgical facts, the prosthetic supply, and the postoperative outcome.

## Supplementary Material

Supplemental Material

## Supplementary data

Table 1 is available as supplementary data in the online version of this article, http://dx.doi.org/10.1080/17453674.2020.1756101
