# Supplementary material for: SwedeAmp—the Swedish Amputation and Prosthetics Registry: 8-year data on 5762 patients with lower limb amputation show sex differences in amputation level and in patient-reported outcome
Source: Acta Orthop. 2020 Apr 22;91(4):464–70. doi: 10.1080/17453674.2020.1756101 (PMC8023884; doi:10.1080/17453674.2020.1756101)
Supplement: Supplemental Material [file IORT_A_1756101_SM7012.pdf]

Supplementary data

Table 1. SwedeAmp forms

Forms	Example of information included
**F1 Basic patient and amputation data**	
	A new F1 has to be registered for every new surgical procedure
	• Name, gender, and the national personal identification number
	• Date of surgery, side, and level of the current amputation surgery
**F2 Amputation data**	
	• Type of procedure (primary amputation, re-amputation to a more proximal level, revision within the same classified amputation level)
	• Operating hospital
	• Underlying diagnosis and immediate cause for amputation
	• Surgical flap technique, parenteral nutrition, antibiotics, and low-molecular heparin
	• Comorbidities, smoking habits, and ability to walk prior to amputation
**F3 Prosthetic supply**	
	• Type of prosthesis (first prosthesis for this level, new socket, or new prosthesis)
	• Type of socket, suspension method, foot and knee component
	• Residual limb problems
	• Date of fitting of the first prosthesis for this amputation
**F4 Baseline data**	
	Baseline patient-reported data describe the patient's situation prior to the impairment leading to amputation
	• Type of accommodation (home with or without assistance or nursing home)
	• Use of walking-aids and/or wheelchair
	• The Locomotor Capability Index-5L (LCI-5L)
**F5 Outcome data**	
	Follow-up and patient-reported data 6, 12, and 24 months after final amputation level
	• *Date of training s*tart with the first prosthesis
	• Returned to the same accommodation as prior to amputation (yes/no)
	• Independently donning and doffing the prosthesis (yes/no)
	• Prosthetic Use Score (0–100)
	• Socket Comfort Score (0–10)
	• LCI-5L (0–56)
	• Use of walking-aids and/or wheelchair
	• Phantom limb pain and residual limb pain
	• Perception of the current general situation as an amputee (very good/good/average/bad/very bad)
	• EQ-5D-5L index
	• Timed-Up-and-Go test (TUG)
**F6 Gait data**	
	• Temporal gait parameters and gait symmetry
	• TUG test and 2-minute walk test
